# Literary Notes

**Published:** 1901-02

**Authors:** 


### LITERARY NOTES.

---

THE *Electrical Review*, of New York, devotes its issue for January 12, 1901, to a review of the history of electrical development during the nineteenth century. Every branch of the field of electrical invention and endeavor is covered in twenty-one special articles by eminent experts. This historical number is embellished with seventy-three portraits of electrical engineers and inventors, whose work has assisted in the building up of the industry.

---

THE *New York State Journal of Medicine*, published monthly by the New York State Medical Association, has appeared. It will publish the transactions of the association, including original contributions and items of news pertaining to the organisation. The name of the editor does not appear.

---

THE *Northwestern Lancet*, of St. Paul, Minn., appeared at the beginning of the year in a new dress. A change of editors has also taken place, Dr. Alexander J. C Stone, who has conducted it from the beginning gives up the chair to Dr. W. A. Jones. It starts off well under the new management.

---

HERBERT S. STONE & CO., of Chicago, announce that they have in preparation, and will soon issue the following important work: A Text Book of Special Surgery, by Dr. Fronz Koenig, for practitioners and students, translated from the seventh German edition, which has but recently appeared, by Arthur B. Hosmer, M.D., and edited by Christian Fenger, M.D. It is the authorised translation, and will consist of three large octavo volumes on an especially fine grade of plate paper, and each volume will contain in the neighborhood of 300 illustrations.

---

DR. M. HARTWIG, president of the Buffalo Academy of Medicine, has offered a prize of $50.00 for the best paper contributed by any member to the Academy during his term of office.

To govern the competitors for this prize the following rules have been adopted by the committee of award:

1. Papers entered for competition must be type-written or printed, and four copies of each paper must be submitted to the committee.

2. All papers must be in the hands of the committee, of which Dr. J. W. Grosvenor, 118 Plymouth Ave., is chairman, on or before July 1, 1901.

3. The announcement of the successful competitor will be made at the first meeting of the Academy after the coming summer vacation. J. W. Grosvenor, B. G. Long, S. Y. Howell, committee.

